# Methanol Partial Oxidation on Cu(111) and PtCu(111) Single-Atom Alloy Surfaces: Effect of Surface Oxygen Coverage on Selectivity

**DOI:** 10.1021/acs.jpcc.6c01011

**Published:** 2026-06-12

**Authors:** Vinita Lal, Maggie Rickman, Jean-Sabin McEwen, Iradwikanari Waluyo, E. Charles H. Sykes

**Affiliations:** † Department of Chemistry, 1810 Tufts University, Medford, Massachusetts 02155, United States; ‡ The Gene and Linda Voiland School of Chemical Engineering and Bioengineering, 6760 Washington State University, Pullman, Washington 99164, United States; § National Synchrotron Light Source II, 8099 Brookhaven National Laboratory, Upton, New York 11973, United States; ∥ Department of Physics and Astronomy, Washington State University, Pullman, Washington 99164, United; ⊥ Department of Chemistry, Washington State University, Pullman, Washington 99164, United States; # Institute for Integrated Catalysis, Pacific Northwest National Laboratory, Richland, Washington 99352, United States; ∇ Department of Biological Systems Engineering, Washington State University, Pullman, Washington 99164, United States

## Abstract

The selective oxidation of methanol to formaldehyde on Cu surfaces is an important and well-studied reaction. However, a systematic analysis of product selectivity as a function of oxygen coverage on Cu(111) and Cu-based single-atom alloys (SAAs) has not been previously reported. In this work, we present a comprehensive investigation of deuterated methanol (CD_3_OH) partial oxidation on Cu(111) and 1% PtCu(111) SAA surfaces as a function of preadsorbed oxygen coverage. Temperature-programmed desorption (TPD) and X-ray photoelectron spectroscopy (XPS) reveal that isolated Pt atoms inhibit the initial surface oxidation of Cu(111) under low oxygen exposures. Despite this difference in oxidation kinetics, the product selectivity remains largely unaffected: on both Cu(111) and PtCu(111), formaldehyde (CD_2_O) is the predominant partial oxidation product over a broad range of oxygen coverages. The selectivity toward formaldehyde peaks at intermediate oxygen coverages (∼0.3 monolayers, ML), indicating the existence of an optimal oxygen loading for partial oxidation. Notably, the similar product selectivities on Cu(111) and PtCu(111) over a range of surface oxygen coverage indicate that Pt single atoms do not significantly alter the reaction pathway or shift the optimal oxygen coverage for formaldehyde formation. Control experiments confirm that Cu(111) is unreactive toward methanol in the absence of oxygen, while PtCu(111) surfaces produce a small amount of formaldehyde even when oxygen is not preadsorbed, indicating that isolated Pt atoms facilitate O–H activation at below 150 K, leading to H_2_ desorption, followed by C–D activation at higher temperatures (∼350 K). Density functional theory (DFT)-based calculations show that Pt atoms increase the O_2_ dissociation barrier relative to Cu(111), consistent with the observed inhibition of oxidation at low exposures. Overall, this work provides the first detailed selectivity map for methanol oxidation on oxidized Cu(111) and PtCu(111) SAA surfaces. By linking classical mechanistic insights—such as methoxy- and formate-mediated pathways—with single-atom alloy catalyst design, this work demonstrates that while Pt substitution modulates the oxidation kinetics and oxygen binding, the overall selectivity toward formaldehyde is governed primarily by oxygen coverage. These findings underscore the potential of isolated dopants to tune surface oxidation behavior without compromising the intrinsic partial oxidation selectivity of copper-based catalysts.

## Graphical abstract

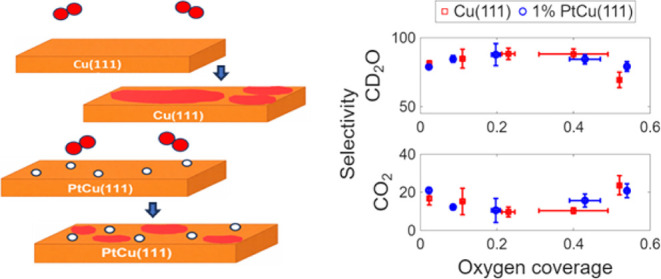

## Introduction

Methanol oxidation to formaldehyde is an important model reaction in heterogeneous catalysis that has been extensively studied on copper surfaces.^1–11^ Early ultrahigh vacuum (UHV) studies established that on Cu single crystals, methanol interacts weakly with clean metal surfaces but readily dissociates in the presence of preadsorbed oxygen to form the methoxy (CH_3_O) intermediate, which subsequently decomposes to release formaldehyde (CH_2_O).^12–24^ In this process, oxygen acts as a hydrogen abstractor, removing the hydroxyl hydrogen from methanol to produce methoxy and water, thereby initiating the selective partial oxidation pathway. While methoxy formation is negligible on flat Cu(111) under UHV conditions, more open facets such as Cu(110) and Cu(100), as well as defect or step sites, can promote limited methanol dissociation even in the absence of oxygen, although the presence of oxygen markedly enhances overall reactivity. Pioneering work by Russell et al. on Cu(111) with preadsorbed oxygen demonstrated that the primary reaction pathway produces formaldehyde via surface methoxy, while a minor decomposition pathway yields CO_2_, likely through a formate (HCOO) intermediate.^15^ This total oxidation channel is promoted by higher oxygen coverages and is fundamentally limited by the high thermal stability of the formate intermediate, which only undergoes decarboxylation to CO_2_ and H_2_ at temperatures significantly higher than those required for selective formaldehyde production (∼470–490 K).^9,11–13,15,25^ Excessive oxygen coverages tend to promote methoxy to formate and further conversion to CO_2_ but can also passivate the surface by blocking adjacent Cu sites required for methoxy formation and decomposition, thereby suppressing both products. Conversely, if the oxygen coverage is too low, methanol cannot activate and desorbs molecularly. Consequently, an intermediate oxygen coverage maximizes formaldehyde yield—a concept that has been demonstrated and discussed extensively on the Cu(111) surface in earlier UHV studies.^15,18,19^

Despite decades of mechanistic insight, the quantitative relationship between surface oxygen coverage and product selectivity remains incomplete. Prior studies have mapped formaldehyde yields as a function of oxygen coverage on Cu(111), yet no studies have systematically determined how the CO_2_ selectivity evolves with oxygen coverage. Such an understanding is crucial for bridging surface science and applied catalysis, since industrial methanol-to-formaldehyde processes on Ag or Cu operate under variable oxygen partial pressures where the surface exists in a dynamic, partially oxidized state. Moreover, the effect of dilute alloying or single-atom modification on the selectivity for partial or total oxidation is largely unexplored. Understanding how small changes in surface composition influence oxidation kinetics and reaction selectivity should provide mechanistic guidance for the catalyst design.

An emerging strategy to tune surface oxidation chemistry is through the incorporation of isolated dopant atoms into a host metal, forming single-atom alloys (SAAs).^26–41^ In these systems, a catalytically active dopant is atomically dispersed in a more inert host, creating well-defined single-atom sites that are active and selective for a broad range of reactions, including hydrogenation,^41–58^ dehydrogenation,^28,38,58–69^ and coupling reactions.^28,33,58,70–74^ In terms of alcohol chemistry, for example, PtCu SAA exhibits enhanced dehydrogenation of ethanol to acetaldehyde, whereby single Pt atoms lower both the O–H bond and C–H bond activation barriers that limit reactivity on Cu(111).^74,75^ To date, the majority of SAA studies have focused on reducing environments; therefore, their behavior under oxidizing conditions—where both metallic and oxidized surface phases may coexist—remains underexplored.^76^

In this work, we address this issue by investigating how isolated Pt dopants influence oxygen-mediated methanol oxidation on Cu. Using a model 1% PtCu(111) SAA, where Pt atoms substitute into the Cu(111) surface,^77^ we quantify methanol oxidation selectivity as a function of surface oxygen coverage and compare it directly to pristine Cu(111). By combining temperature-programmed desorption (TPD) experiments with *in situ* X-ray photoelectron spectroscopy (XPS) and density functional theory (DFT) calculations, we show that Pt subtly modifies the oxygen adsorption kinetics—retarding the initial oxidation of Cu(111)—but does not significantly shift the optimum conditions for formaldehyde vs CO_2_ formation. The formaldehyde selectivity as a function of oxygen coverage is nearly identical on Cu(111) and PtCu(111), demonstrating that surface oxygen coverage, rather than the dopant, dominates the reaction selectivity. These results provide the first oxygen coverage-dependent selectivity map for methanol oxidation on a SAA surface and offer new mechanistic insight into how isolated dopants modulate oxidation kinetics without altering intrinsic selectivity.

## Materials and Methods

### Temperature-Programmed Desorption (TPD)

All TPD experiments were carried out in a UHV chamber supported with a turbomolecular pump to attain UHV conditions and a titanium sublimation pump to maintain those conditions. The base pressure in the chamber was ∼7.5 × 10^–11^ Torr. The Cu(111) crystal was mounted to a manipulator through 0.25 mm thick Ta wires. The crystal was resistively heated via the Ta wires using a DC power supply and was cooled to 87 K via a liquid nitrogen cryostat. The crystal surface was cleaned using repeated cycles of Ar^+^ ion bombardment using a hot filament sputter source from RBD Instruments and thermal annealing to 723 K. The temperature was measured by a type K thermocouple welded to the back of the crystal. ^16^O_2_ (99.999%, Praxair) was dosed at 400 K using a precision leak valve (MDC Precision), and the crystal was moved within 3 in. of the leak valve while facing in its direction. A saturated 0.586 ML oxygen coverage was assumed to correspond to the “29” Cu_*x*_O structure.^78^ Pt was deposited at 383 K and 10 nA flux by electron bombardment evaporation of a high-purity Pt rod.^79^

### X-ray Photoelectron Spectroscopy (XPS)

XPS experiments were carried out at the *In situ* and Operando Soft X-ray spectroscopy (IOS, 23-ID-2) beamline at the National Synchrotron Light Source II (NSLS-II), Brookhaven National Laboratory. A technical description of the beamline and endstation is detailed elsewhere.^80^ The Cu(111) crystal was cleaned by multiple Ar^+^ sputtering (1 keV) and annealing (850 K) cycles until XPS showed that the surface was clean. To prepare PtCu(111) SAA, Pt was deposited onto the Cu(111) surface at 360–380 K using an SPECS EBE-4 electron beam evaporator. O_2_ (Matheson, 99.994%) and CO (Matheson, 99.999%) were dosed using high-precision variable leak valves, and the pressure was read by a hot cathode ion gauge. Photon energies of 710 and 500 eV were used to obtain O 1*s* and C 1*s* spectra, respectively. A Cu oxidation study was performed while maintaining a constant O_2_ pressure at a sample temperature of 400 K, and O 1*s* spectra were continuously acquired every 35 s. The XPS binding energy was referenced to the Fermi edge measured at each photon energy. The same experimental procedure performed without exposure to an X-ray beam yielded the same final result, thus ruling out any possible beam-induced effects.

### Computational Methods

All density functional theory (DFT) simulations were conducted using the Vienna Ab initio Simulation Package (VASP) software.^81,82^ Electron smearing was performed with the Gaussian method with a smearing width of 0.2 eV, and the total energy was extrapolated to 0 K. Geometric optimizations were considered converged when the total energy changed by less than 1 × 10^–4^ eV and interatomic forces were less than 0.03 eV/Å for the ground-state optimizations. The core electrons were described using the projector augmented wave (PAW) method that was released in 2017 ^83,84^ and the valence electrons were modeled using a plane-wave basis set that was expanded to a cutoff energy of 500 eV. The exchange-correlation potential was modeled with the Perdew–Burke–Ernzerhof functional.^85^ Structural visualizations were performed with VESTA.^86^

For adsorption calculations, O_2_ dissociation was carried out on a four-atomic-layer-thick Cu(111) surface with a *p*(8 × 8) supercell in order to minimize the lateral interactions between the O_2_ molecules. The bulk lattice constant was determined to be 3.635 Å, where a 8 × 8 × 8 Monkhorst–Pack grid was used.^87^ Then, the same adsorption calculations were carried out on an SAA PtCu(111) surface. This surface was constructed by substituting one of the Cu atoms in the top layer with a Pt atom. In all geometry optimization calculations, the bottom two layers were fixed at their optimized bulk positions while the top two layers were allowed to fully relax. A vacuum layer of 10 Å and a 1 × 1 × 1 Monkhorst–Pack grid were used to sample the first Brillouin zone for all reaction pathways. We tested the effect of including a dipole layer sheet in our calculations and found that the root-mean-square residual value as compared to the system without a dipole layer correction was 0.004 eV. Since this had a minimal impact, we did not include a dipole layer sheet in our calculations. All minimum energy pathways were obtained with either the Nudged Elastic Band (NEB) method^88^ or the Climbing Image Nudged Elastic Band (CI-NEB) method,^89^ and were optimized until the residual forces on all atoms were smaller than 0.03 eV/Å. The adsorption sites of oxygen atoms were evaluated by calculating the adsorption energy. This was calculated as:

1 $$E_{\mathrm{ads}}=E_{\mathrm{surface}+\mathrm{atoms}}-E_{O_{2}}-E_{\mathrm{surface}}$$

where *E*_surface+atoms_ is the total energy of the oxygen atoms adsorbed on surface, *E*_surface_ is the total energy of the Cu(111) or PtCu(111) surface, and $E_{O_{2}}$ is the energy of an isolated oxygen spin-polarized molecule in the gas phase. In this work, we concentrated our efforts on the peroxo-like $O_{2}^{2-}$ species since it has been established that it binds more strongly to the surface as compared to the superoxo-like $O_{2}^{-}$ species.^90^ Since it has been established that the peroxo-like species do not have a net magnetic moment associated with them, our calculations were not spin polarized. Finally, in calculating the adsorption energies, we did not take zero-point energy contributions into account since our test calculations on Cu(111) show that they change the adsorption energy of chemisorbed O_2_ by 0.12 eV and that of dissociated O_2_ by 0.15 eV. As such, these contributions were neglected since the resulting change of 0.03 eV between chemisorbed O_2_ and dissociated O_2_ configurations will likely not impact the dissociation energetics between these two states.

## Results and Discussion

To establish how methanol oxidation proceeds on Cu(111) and on the PtCu(111) SAA, we performed temperature-programmed methanol uptake experiments on both surfaces preoxidized by dosing 6.75 Langmuir (L, 1 L = 1 × 10^-6^ Torr·s)­O_2_ at 400 K. After oxidation, the samples were cooled to 87 K for CD_3_OH adsorption and then heated linearly at 1.0 K/s to induce desorption or reaction. Figures 1A and 1B show representative TPD profiles obtained from oxidized Cu(111) and 1% PtCu(111) SAA, respectively. On both surfaces, formaldehyde (CD_2_O) is the predominant carbon-containing product, accompanied by smaller amounts of CO_2_. A trace amount of CO was detected, which was attributed to the fragment from formaldehyde and CO_2_. On PtCu(111), the CO desorption peak at ∼350 K corresponded to CO that had adsorbed from the UHV background on Pt sites.^91^ A minor pathway of recombinative methanol (CD_3_OD) desorption at the same temperature as CD_2_O was also observed on both surfaces. The similar amounts of products evolving from both samples demonstrate that in the presence of oxygen, Pt incorporation does not introduce new reaction pathways. Therefore, it is expected that methanol activation proceeds through the same sequence of steps established for Cu surfaces: abstraction of the hydroxyl hydrogen by adsorbed oxygen to form surface methoxy, followed either by decomposition to formaldehyde or further oxidation to formate and CO_2_.

**Figure 1:**
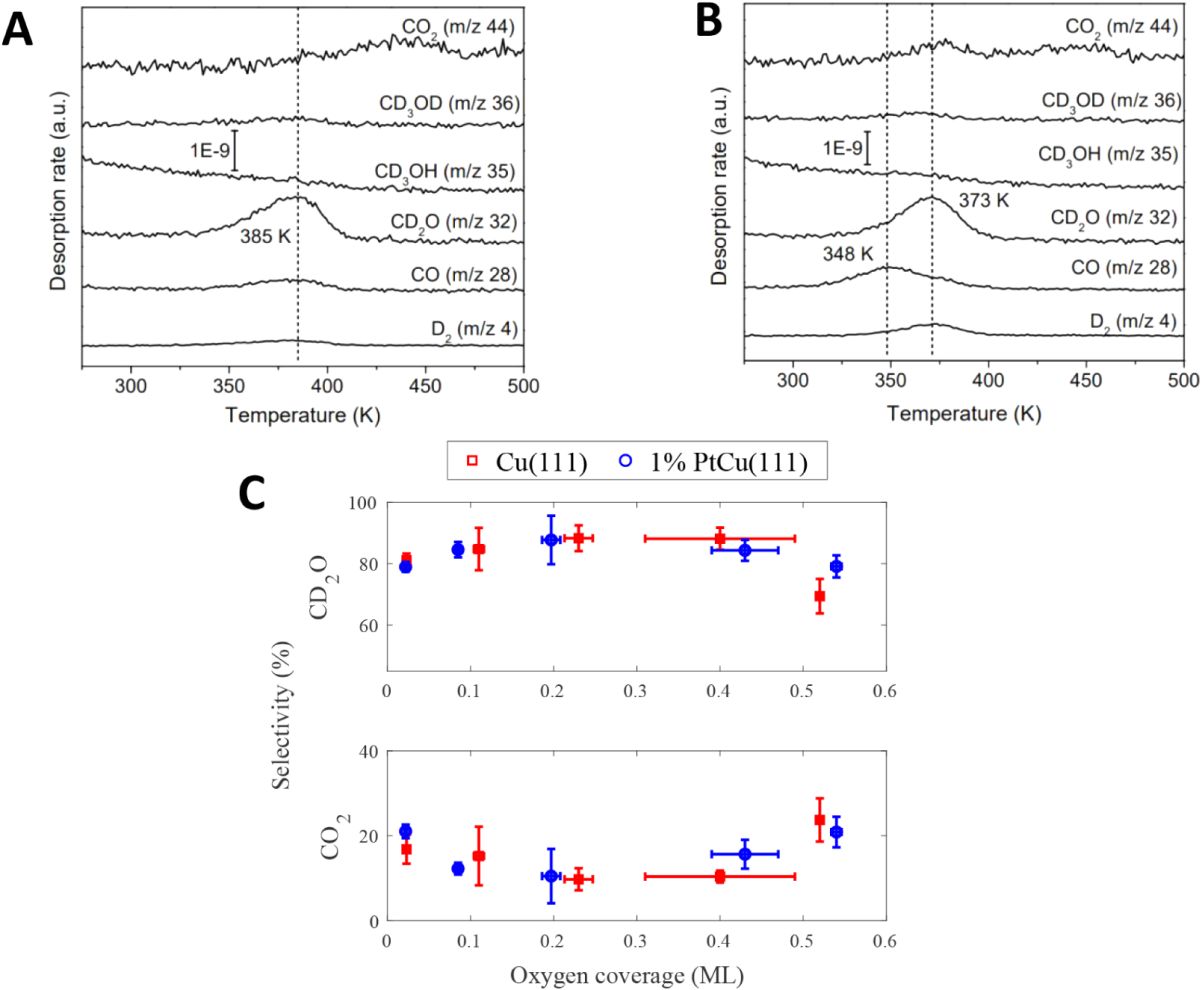
Methanol oxidation on Cu(111) and PtCu(111) SAA samples. Representative TPD traces for (A) oxidized Cu(111) and (B) oxidized 1% PtCu(111) using ∼0.02 ML O reacting with deuterated methanol. (C) Selectivity toward products from deuterated methanol oxidation on Cu(111) and 1% PtCu(111) as a function of oxygen coverage. A saturated 0.586 ML oxygen coverage was assumed to correspond to the “29” Cu*_x_* O structure.^78^

Many TPD experiments were performed as a function of oxygen coverage, and the quantitative product selectivity is summarized in Figure 1C. The surface oxygen coverage was determined by integrating the total yield of CD_2_O and CO_2_ obtained from methanol titration. Previous studies on Cu(111) quantified oxygen coverage using only CD_2_O yield.^92–94^ However, based on the kinetic framework established by Russell et al.,^15^ both products must be considered to properly account for total oxygen consumption. In the accepted mechanism for methanol oxidation on Cu(111), two formaldehyde molecules correspond to the removal of one surface oxygen atom, whereas the production of two CO_2_ molecules via the overoxidation channel consumes three surface O atoms in total, as shown below:

2 $$2CD_{3}OH+O\to 2CD_{2}O+H_{2}O+D_{2},\mathrm{Partial oxidation pathway}$$

3 $$2CD_{3}OH+3O\to 2CO_{2}+H_{2}O+3D_{2},\mathrm{Total oxidation pathway}$$

Using this stoichiometric relationship, the initial oxygen coverage was calculated from the CD_2_O and CO_2_ yields for both surfaces. The results are shown in Figure S1. Product selectivity was defined as the ratio of the individual product yield (CD_2_O or CO_2_) to the total carbon-containing products (CD_2_O + CO_2_).

Figure 1C illustrates the dependence of methanol oxidation selectivity on preadsorbed oxygen coverage for both Cu(111) and 1% PtCu(111). With very low oxygen coverage (<0.1 ML), CD_2_O accounts for approximately 80% of the carbon-containing products, while CO_2_ remains a minor product. As the oxygen coverage increases, the CD_2_O selectivity initially increases and reaches a maximum of ∼90% at intermediate oxygen coverages of ∼0.25–0.30 ML, defining the optimal regime for methanol partial oxidation.^15^ At higher oxygen coverages, the CD_2_O selectivity decreases gradually, accompanied by a corresponding increase in CO_2_ selectivity. This trend indicates that increasing surface oxygen favors the overoxidation pathway relative to formaldehyde formation.

Importantly, the selectivity curves for Cu(111) and PtCu(111) overlap within experimental uncertainty across the entire oxygen-coverage range. The oxygen coverage corresponding to maximum CD_2_O selectivity and the subsequent rise in CO_2_ selectivity are nearly identical on both surfaces. These results demonstrate that the branching between partial oxidation (CD_2_O) and total oxidation (CO_2_) is determined solely by oxygen coverage and not by the presence of Pt dopants.

Although Pt does not alter the oxygen-coverage-dependent selectivity trend (Figure 1C), differences are observed in the product desorption temperatures (Figures S2–S3; representative low oxygen coverage TPD traces shown in Figure 1A and 1B). On Cu(111), formaldehyde desorbs between 384 and 425 K, with the peak temperature increasing with increasing oxygen coverage (Figure S2A-top TPD traces). On 1% PtCu(111), the CD_2_O peak appears between 370 and 410 K, i.e., approximately 10–15 K lower than on Cu(111) at comparable coverages (Figure S2A-bottom). A similar trend is observed for CO_2_. For the same representative coverage series as shown in Figure S2A, the CO_2_ peak on PtCu(111) is shifted to lower temperatures relative to Cu(111), with the largest differences occurring at lower oxygen coverages and diminishing at higher oxygen coverages. Notably, at saturated oxygen coverage (∼0.5 ML), the CO_2_ peak temperature in the first TPD during sequential methanol titration is essentially the same on both surfaces (∼415 K on Cu(111) vs ∼413 K on PtCu(111) (Figure S3). However, as the surface oxygen is progressively consumed during titration, the CO_2_ peak on PtCu(111) shifts downward to ∼400 K. Thus, while the initial CO_2_ desorption temperatures are comparable at high oxygen coverage, the PtCu(111) surface exhibits lower CO_2_ desorption temperatures as oxygen coverage decreases. These shifts in desorption temperature are consistent with the formate-mediated overoxidation mechanism established for Cu surfaces, indicating that isolated Pt atoms facilitate C–D bond activation and may lower the barrier for formate decomposition to CO_2_.^95^

When the surfaces are saturated by oxygen, methanol reactivity is limited due to the reduced availability of metal oxide interface sites.^94^ As methanol is deposited and desorbed repeatedly during titration experiments, the progressive removal of surface oxygen increases the accessibility of isolated Pt atoms embedded in the Cu surface. This increased accessibility likely contributes to the lower CO_2_ desorption temperatures observed on PtCu(111) relative to Cu(111).

The concurrent desorption of CD_3_OD and D_2_ alongside that of CD_2_O on both surfaces implies a common methoxy intermediate. Specifically, when a methoxy species undergoes C–D cleavage, the following steps can occur: (i) the methoxy can desorb as CD_2_O, (ii) the D atom can combine with another methoxy to form methanol (desorbing as CD_3_OD), and (iii) the D atoms can couple and desorb as D_2_. The simultaneous desorption of CD_3_OD, D_2_, and CD_2_O thus confirms that they share the methoxy origin, whereas the higher-temperature CO_2_ peak indicates that an additional step—likely formation and decomposition of a formate intermediate—is required for the total oxidation pathway.^2–6,8–16,25,96–99^

Having established that Pt does not alter the oxygen-coverage–dependent selectivity of methanol oxidation, we examined how Pt influences the initial oxidation of Cu(111). Figure 2A shows representative XPS O 1*s* spectra collected during the oxidation of Cu(111) and PtCu(111) at 400 K under 5 × 10^–8^ Torr O_2_. The intensity of this peak increases progressively with O_2_ exposure time, indicating accumulation of atomic oxygen on the surface. After ∼40 min of exposure, the O 1*s* signal for PtCu(111) is approximately 25% lower than that of Cu(111), consistent with a slightly slower oxidation rate on the PtCu(111) surface. Interestingly, this contrasts with 1% Rh doping of Cu(111), which accelerates the initial oxidation of Cu(111) ∼9-fold.^92^ The representative XPS spectra presented in Figure 2A illustrate the slightly slower oxidation of PtCu(111) and confirm that the same oxygen species form on both surfaces throughout the oxidation process. No additional O 1*s* components at higher binding energy were detected, indicating no hydroxyl or subsurface contributions under these conditions.

**Figure 2:**
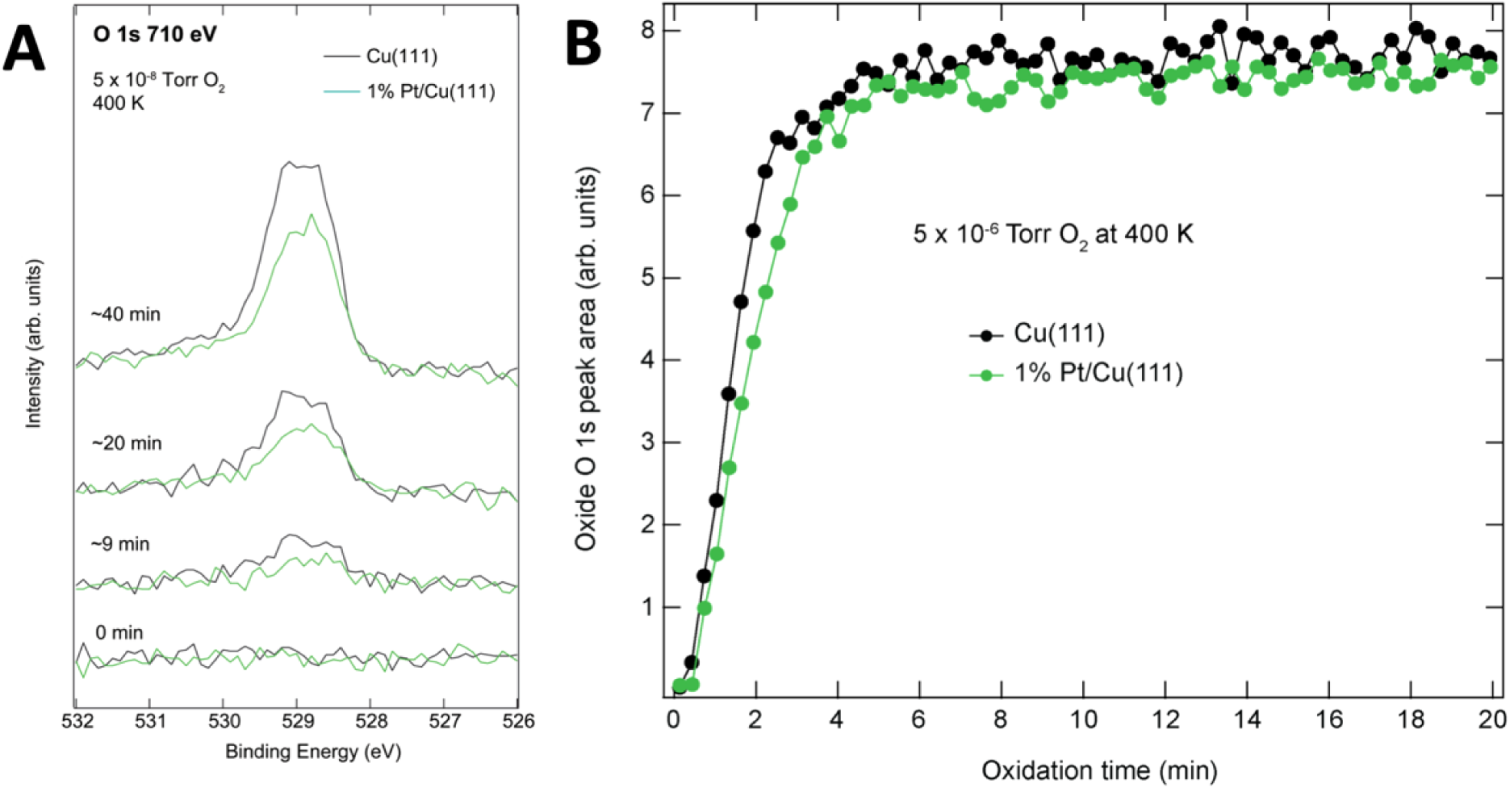
XPS data of the oxidation of Cu(111) and PtCu(111) SAA. (A) Representative O 1*s* spectra during exposure to 5 × 10^–8^ Torr of O_2_ at 400 K for the two surfaces. (B) Plots of O 1*s* XPS peak area during extended oxidation with exposure to 5 × 10^–6^ Torr of O_2_ at 400 K for Cu(111) (black circles) and 1% PtCu(111) (green circles).

Figure 2B shows the extended oxidation behavior of both surfaces. After ∼6 min of O_2_ exposure at 5 × 10^–6^ Torr, the O 1*s* intensities for Cu(111) and PtCu(111) converge, demonstrating that both surfaces reach essentially the same oxygen coverage. At longer times and higher O_2_ pressures, both surfaces exhibit stable O 1*s* intensities and constant O 1*s* binding energies, confirming that Pt does not alter the chemical environment or stoichiometry of the oxidized Cu surface. In contrast to the aforementioned RhCu(111), which enhances initial oxidation but later suppresses deeper oxide growth,^92^ Pt displays only a slight inhibitory effect, slowing the initial O_2_ dissociation without changing the terminal oxygen coverage.

The oxygen coverage calculated using TPD shown in Figure S1 is in reasonable agreement with the XPS-derived coverage trends. These differences likely arise from the distinct measurement approaches—TPD quantifies oxygen removal through methanol titration, which has error associated with the quantification of the different reaction products, whereas XPS directly probes the surface O 1*s* peak intensity. However, both methods consistently indicate the inhibitory effect of Pt in the initial stages of surface oxidation and show that saturated oxygen coverages are the same on Cu(111) and PtCu(111).

In order to understand the origin of the slightly different rates of initial oxidation of Cu and the PtCu SAA, we performed DFT calculations. Figure 3 compares the minimum energy pathway for O_2_ adsorption and dissociation on PtCu(111) for two different scenarios—one where O_2_ is close to the Pt dopant (Figure 3A) and one where O_2_ is far from the Pt dopant (Figure 3B). It is apparent from this data that when O_2_ adsorbs far from the Pt atom, the adsorption energy of the O_2_ molecule is essentially that of O_2_ on Cu(111) and proceeds downhill in energy. In contrast, when O_2_ adsorbs near the isolated Pt atom, the initial interaction with Pt is repulsive, as reflected by a less favorable adsorption energy as compared to when O_2_ adsorbs far from the Pt site. Although dissociation is still possible, the pathway is less stabilized overall, and the barrier for O–O scission is slightly higher. This demonstrates that the Pt atom does not provide a low-barrier entry point for O_2_ activation; instead, it locally destabilizes molecular adsorption of O_2_ in the region around the Pt atom, hindering efficient uptake of oxygen on the surface. These results are consistent with our previous work in which we found that surface O was destabilized in the vicinity of Pt as compared to surrounding Cu(111) sites.^100^ We note that the adsorption energy of oxygen is more favorable in this work because we used a *p*(8 × 8) unit cell, whereas our previous calculations employed a *p*(4 × 4) unit cell.^100^

**Figure 3:**
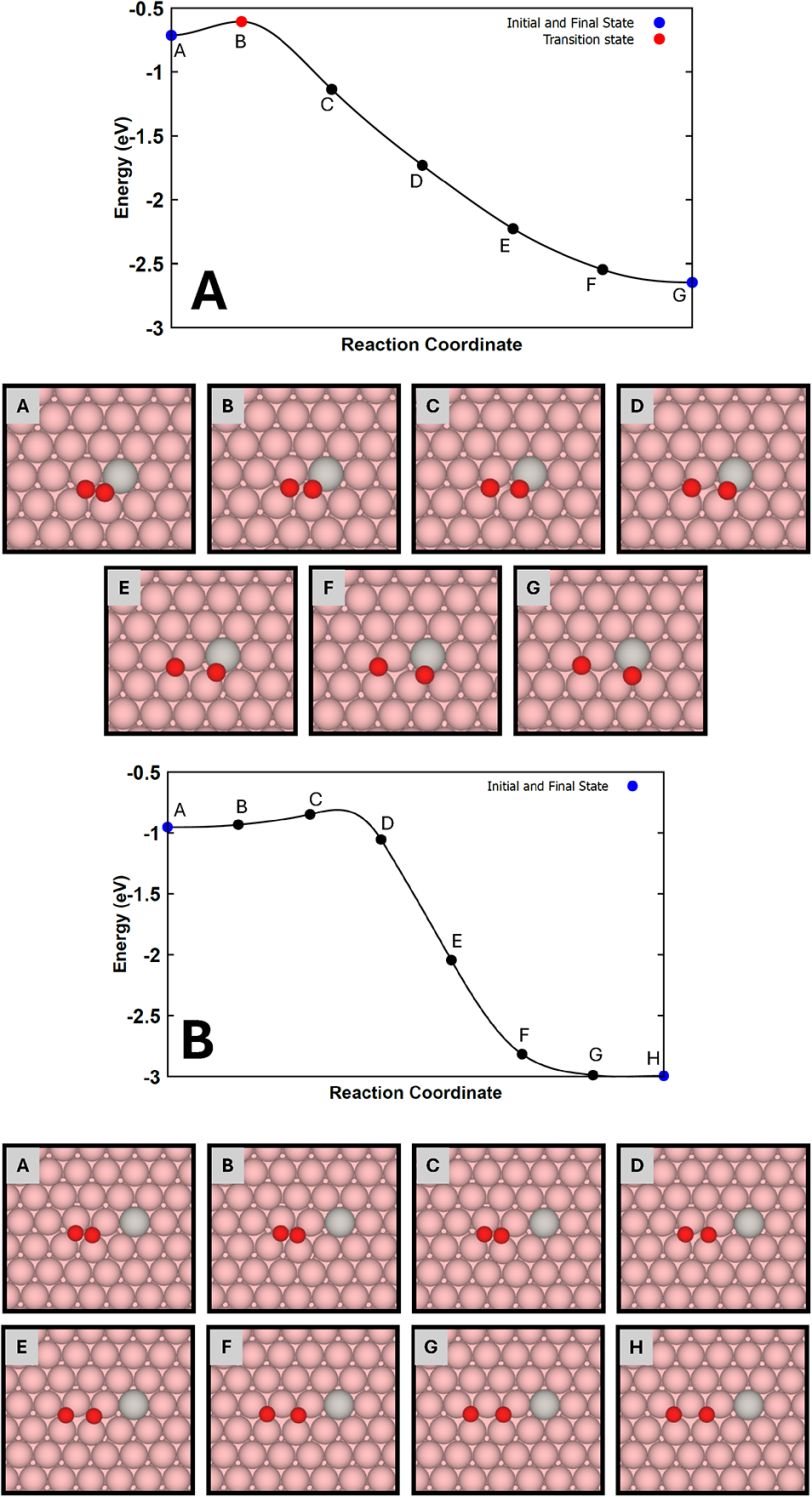
DFT-calculated minimum energy pathway and corresponding configurations for O_2_ dissociation on PtCu(111) SAA surfaces with (A) O_2_ close to the Pt dopant and (B) O_2_ far from the Pt dopant. The Cu, Pt, and O atoms are represented by pink, gray, and red spheres, respectively. This energy scale is with respect to an O_2_ molecule in the gas phase.

During sequential methanol titration experiments, an additional reaction pathway was observed on PtCu(111) that operates even in the absence of preadsorbed surface oxygen. On Cu(111), methanol oxidation occurs only when surface oxygen is present, producing formaldehyde and CO_2_ during the first TPD cycle, as shown in TPD1 of Figure 4A. Once the surface oxygen is fully consumed, subsequent TPD traces are flat, consistent with previous reports that preadsorbed oxygen is essential for methanol oxidation on flat Cu(111) surfaces.^12,15,18–21^ In contrast, the 1% PtCu(111) SAA continues to form a small but constant amount of formaldehyde after the first TPD (Figure 4B). This persistent signal indicates that the isolated Pt atoms embedded in Cu(111) can activate both O–H and C–H bonds in methanol, forming methoxy intermediates even without oxygen present. Repeated TPD cycles up to 573 K show that both the formaldehyde yield and Pt coverage remain constant. The constant Pt coverage remaining after each methanol TPD was determined by the adsorption of CO, which desorbs at low temperatures (<200 K) from Cu(111) vs ∼350 K from the embedded Pt atoms, confirming that approximately half the Pt dopants initially deposited (1%) are stable and catalytically accessible under these reaction conditions. While this low-coverage dehydrogenation route is not dominant under oxidizing conditions, it illustrates how single-atom dopants can introduce new, low-barrier C–H and O–H activation pathways unavailable on flat Cu(111) surfaces.

**Figure 4:**
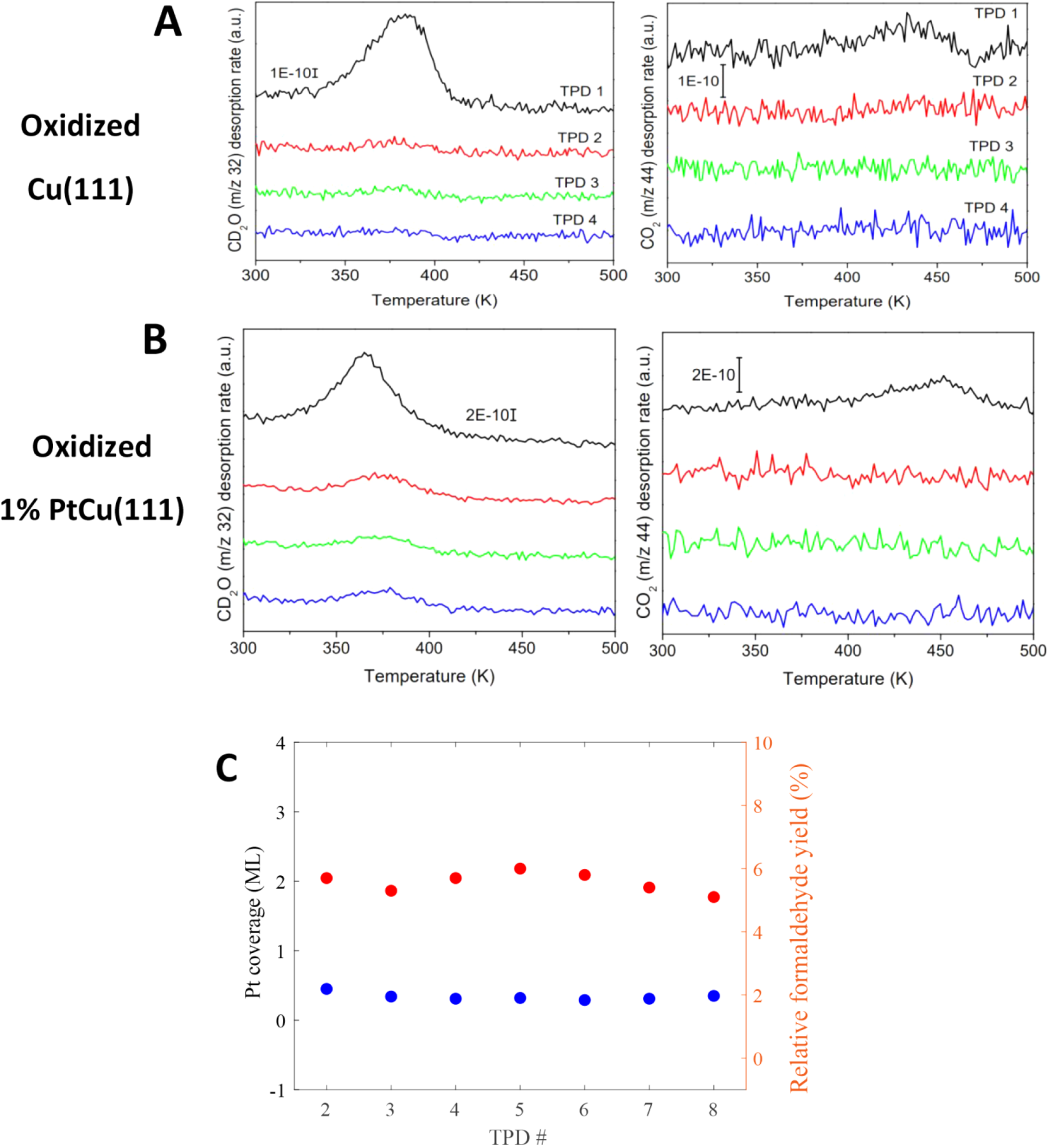
Sequential methanol TPDs showing formaldehyde and CO_2_ production on (A) clean Cu(111) and (B) 1% PtCu SAA after 6.75 L O_2_ exposure at 400 K. The reaction ceases after 1 cycle on Cu(111), but the presence of Pt enables a small amount of methanol to formaldehyde conversion even when the surface oxygen is depleted. (C) Repeated TPD cycles (83 K–573 K) demonstrate a constant formaldehyde yield (red points) and unchanged Pt coverage (blue points) as determined by CO titration,^29,62^ indicating that the Pt dopants remain stable in the surface and retain their activity over multiple reaction cycles.

## Conclusion

This study provides the first comprehensive, oxygen coverage-dependent selectivity map for methanol partial oxidation on Cu(111) and 1% PtCu(111) single-atom alloy surfaces. By combining TPD, XPS, and DFT calculations, we demonstrate that surface oxygen coverage—rather than the presence of isolated Pt dopants—governs the selectivity branching between methanol partial oxidation to formaldehyde and total oxidation to CO_2_. Formaldehyde selectivity reaches a maximum of ∼90% at intermediate oxygen coverages (∼0.25–0.30 ML) on both surfaces, confirming that an optimal oxygen loading is required to balance methoxy formation and overoxidation pathways. While Pt incorporation slightly inhibits initial O_2_ dissociation and slows early-stage Cu oxidation kinetics, it does not shift the oxygen coverage window for maximum formaldehyde selectivity. Instead, Pt subtly lowers reaction temperatures for product evolution and enables limited methanol dehydrogenation even in the absence of preadsorbed oxygen, highlighting its ability to facilitate O–H and C–H activation. These findings demonstrate that isolated dopants can tune oxidation kinetics without disrupting the intrinsic selectivity trends governed by surface oxygen coverage, providing a strategy to adjust oxidation behavior while preserving the desirable partial oxidation selectivity of copper-based catalysts.

## Supplementary Material



## Data Availability

Data will be made available on request.

## Notes

TPD-derived oxygen coverages and additional TPD data illustrating oxygen coverage-dependent product desorption temperatures and sequential oxygen titration behavior on Cu(111) and PtCu(111) (PDF)
