# Supplementary material for: Methanol Partial Oxidation on Cu(111) and PtCu(111) Single-Atom Alloy Surfaces: Effect of Surface Oxygen Coverage on Selectivity
Source: J Phys Chem C Nanomater Interfaces. 2026 Jun 12;130(25):8706–15. doi: 10.1021/acs.jpcc.6c01011 (PMC13312450; doi:10.1021/acs.jpcc.6c01011)
Supplement: Supplementary file 1 [file jp6c01011_si_001.pdf]

Supporting Information

Methanol Partial Oxidation on Cu(111) and PtCu(111) Single-Atom Alloy Surfaces: Effect of Surface Oxygen Coverage on Selectivity

Vinita Lal[1], Maggie Rickman[2], Jean-Sabin McEwen*[2,4,5,6,7], Iradwikanari Waluyo*[3], E. Charles H. Sykes*[1]

[1] Department of Chemistry, Tufts University, Medford, Massachusetts 02155, United States

[2] The Gene and Linda Voiland School of Chemical Engineering and Bioengineering, Washington State University, WA 99164, United States

[3] National Synchrotron Light Source II, Brookhaven National Laboratory, Upton, NY 11973, United States

[4] Department of Physics and Astronomy, Washington State University, Pullman, WA 99164, United

[5] Department of Chemistry, Washington State University, Pullman, WA 99164, United States

[6] Institute for Integrated Catalysis, Pacific Northwest National Laboratory, Richland, WA 99352, United States

[7] Department of Biological Systems Engineering, Washington State University, Pullman, WA 99164, United States

* Corresponding author: js.mcewen@wsu.edu

* Corresponding author: iwaluyo@bnl.gov

* Corresponding author: charles.sykes@tufts.edu

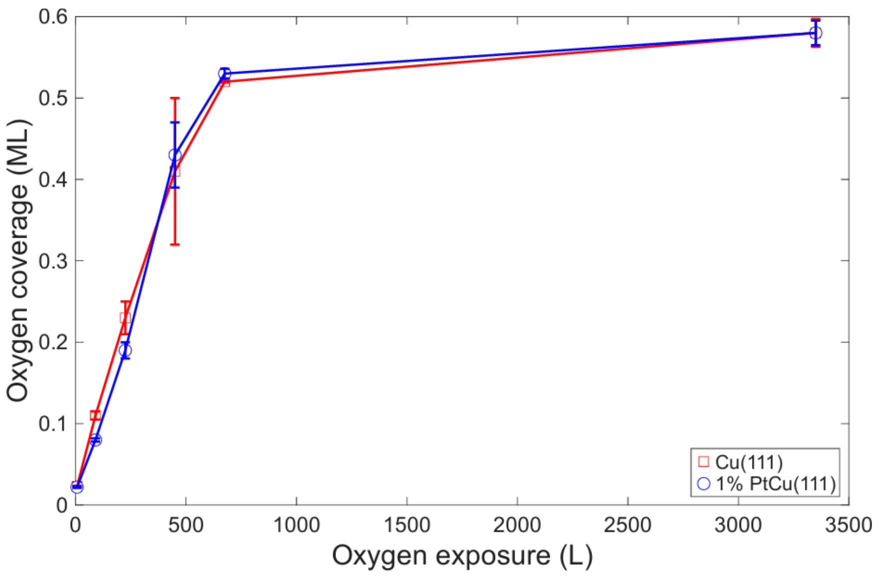

Figure S1: Oxygen coverage determined from methanol titration TPD measurements as a function of O_2 exposure on Cu(111) (red squares) and 1% PtCu(111) (blue circles). Oxygen coverage increases with exposure for both surfaces and approaches ~0.5 - 0.6 ML at high exposures. Error bars reflect uncertainties in coverage determination based on product integration. The similar saturation behavior indicates comparable terminal oxygen coverages on Cu(111) and PtCu(111).

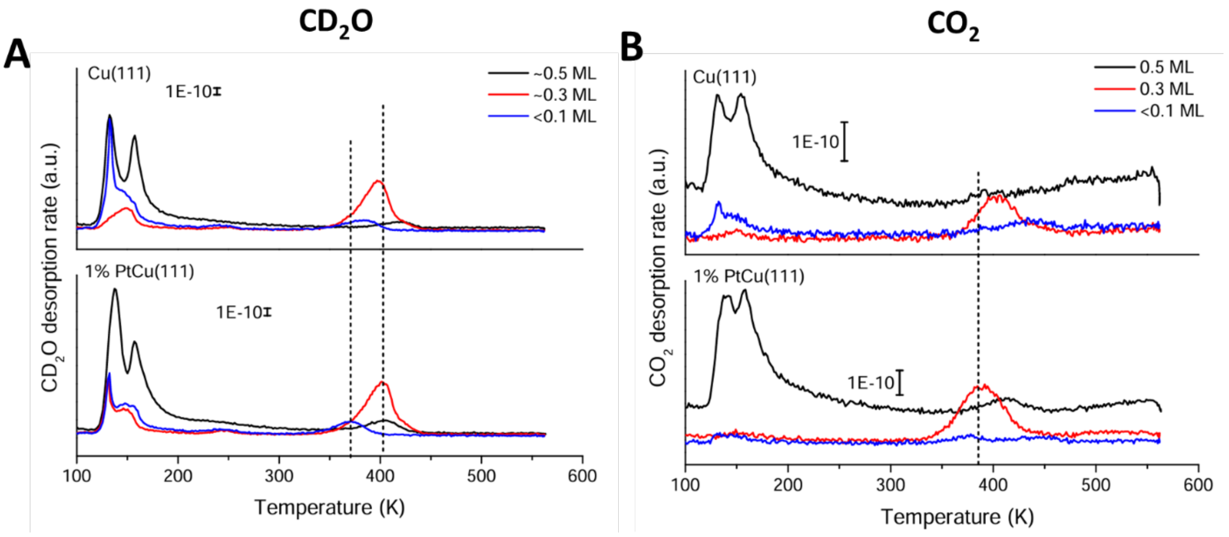

Figure S2: TPD spectra of CD_2O (A) and CO_2 (B) following methanol titration on Cu(111) (top panels) and 1% PtCu(111) (bottom panels) at ~0.5, ~0.3, and <0.1 ML oxygen coverage. Increasing oxygen coverage shifts CD_2O desorption to higher temperature on both surfaces. At comparable coverages, PtCu(111) exhibits slightly lower CD_2O and CO_2 desorption temperatures than Cu(111), with the largest differences observed at lower oxygen coverages.

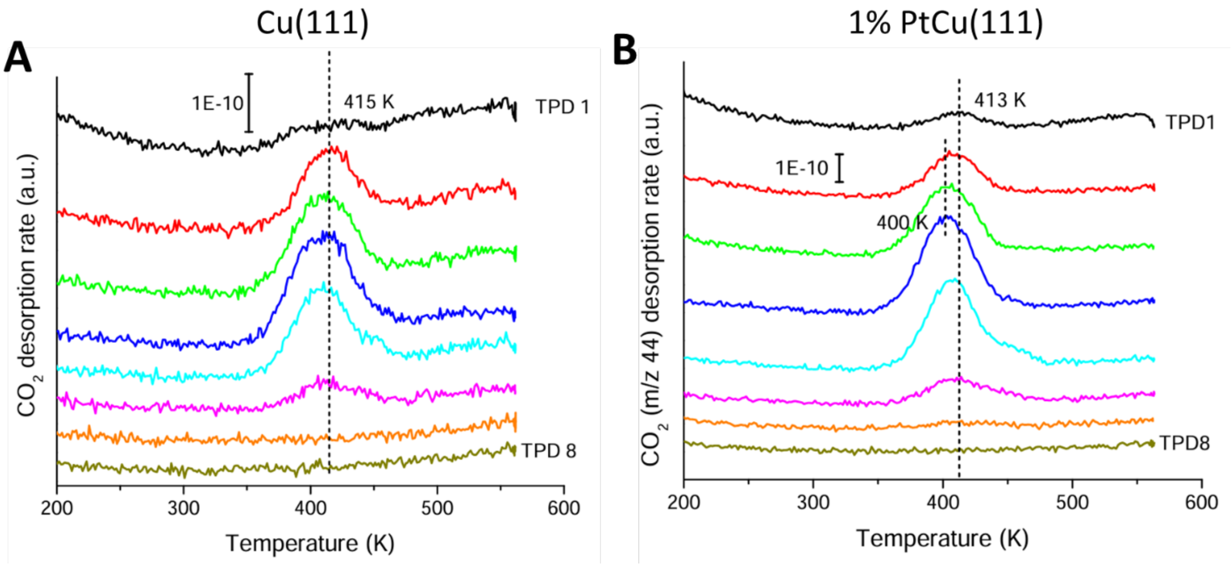

Figure S3: Sequential methanol titration TPD spectra showing CO_2 evolution on Cu(111) (left) and 1% PtCu(111) (right) starting from saturated oxygen coverage (~0.5 ML). TPD1 corresponds to the first methanol exposure, while subsequent traces (TPD2 - TPD8) reflect progressive oxygen consumption. On Cu(111), the primary CO_2 desorption feature remains centered near ~415 K throughout the titration sequence. In contrast, on PtCu(111), the initial CO_2 peak appears at ~413 K and shifts to lower temperature (~400 K) as surface oxygen is progressively removed.

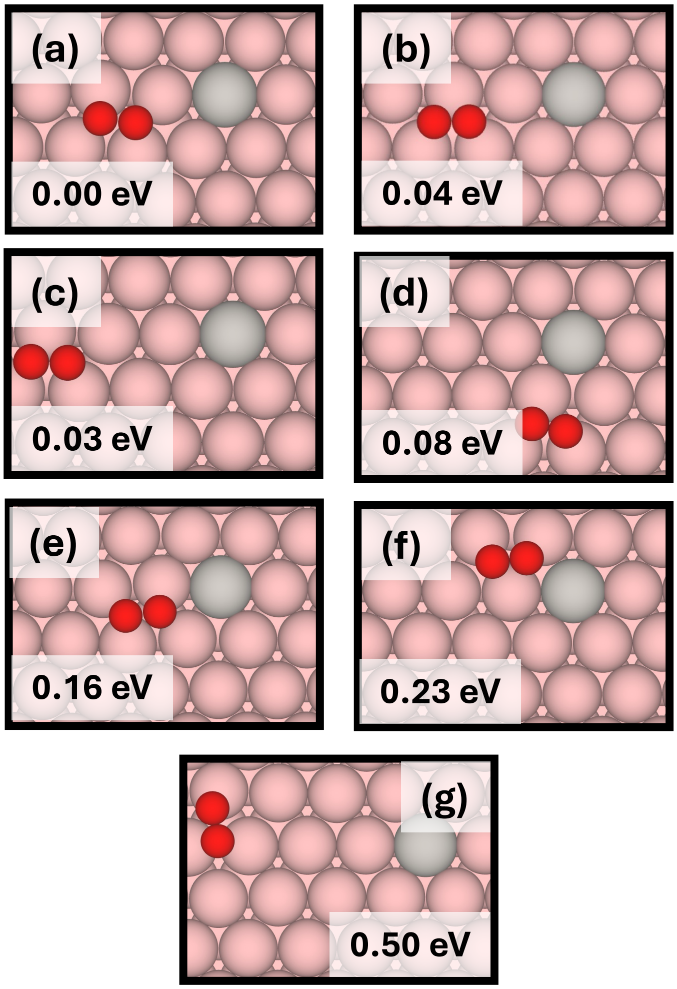

Figure S4: Adsorption energy minima for O_2 on Pt/Cu(111) were used to determine the initial configurations for O_2 dissociation. The starting point for dissociation far from the Pt single-atom alloy (SAA) is shown in (a), while the starting point near the Pt SAA corresponds to the intermediate minimum along the minimum-energy pathway originating from configuration (e). All energies are referenced to the most favorable adsorption site. Red, pink, and grey spheres represent O, Cu, and Pt atoms, respectively.
